# Non-Invasive whole-body detection of complement activation using radionuclide imaging in a mouse model of myocardial ischaemia-reperfusion injury

**DOI:** 10.1038/s41598-017-16387-1

**Published:** 2017-11-23

**Authors:** Ehsan Sharif-Paghaleh, May Lin Yap, Sarah-Lena Puhl, Adam Badar, Julia Baguña Torres, Krisanat Chuamsaamarkkee, Florian Kampmeier, Richard A. Smith, James Clark, Philip J. Blower, Steven Sacks, Gregory E. Mullen

**Affiliations:** 1 Division of Imaging Sciences and Biomedical Engineering, St Thomas’ Hospital, King’s College London, London, UK; 2 MRC Centre for Transplantation, King’s College London, London, UK; 3 Department of Immunology, School of Medicine, Tehran University of Medical Sciences, Tehran, Iran; 4 Cardiovascular Division, Faculty of Life Sciences and Medicine, King’s College London, London, UK

## Abstract

Complement activation is a recognised mediator of myocardial ischaemia-reperfusion-injury (IRI) and cardiomyocytes are a known source of complement proteins including the central component C3, whose activation products can mediate tissue inflammation, cell death and profibrotic signalling. We investigated the potential to detect and quantify the stable covalently bound product C3d by external body imaging, as a marker of complement activation in heart muscle in a murine model of myocardial IRI. We used single-photon-emission-computed-tomography (SPECT) in conjunction with ^99m^Technecium-labelled recombinant complement receptor 2 (^99m^Tc-rCR2), which specifically detects C3d at the site of complement activation. Compared to control imaging with an inactive CR2 mutant (^99m^Tc-K41E CR2) or an irrelevant protein (^99m^Tc-PSMA) or using ^99m^Tc-rCR2 in C3-deficient mice, the use of ^99m^Tc-rCR2 in complement-intact mice gave specific uptake in the reperfused myocardium. The heart to skeletal muscle ratio of ^99m^Tc-rCR2 was significantly higher than in the three control groups. Histological analysis confirmed specific uptake of ^99m^Tc-rCR2. Following therapeutic inhibition of complement C3 activation, we found reduced myocardial uptake of ^99m^Tc-rCR2. We conclude, therefore that ^99m^Tc-rCR2 imaging can be used for non-invasive detection of activated complement and in future could be exploited to quantify the severity of myocardial damage due to complement activation.

## Introduction

Although prompt restoration of myocardial blood flow represents the most effective intervention for patients with acute myocardial infarction (AMI), reperfusion following a period of restricted blood flow can itself be a major cause of cardiac damage, worsening the outcome for patients^1^. This phenomenon, named ischaemia-reperfusion injury (IRI) has a complex pathophysiology in which at least three major factors contribute to tissue injury: *in situ* production of reactive oxygen species (ROS); activation of white blood cells (neutrophils and macrophages); and generation of components of the activated complement (C) cascade^2^. Complement activation induced by IRI can involve three known pathways: the lectin (or mannose-binding lectin) pathway, and the alternative and classical pathways. All of these pathways converge on the complement protein C3. C3 is synthesised by tissue parenchyma as an early response to tissue stress or infection^3^. Large amounts of C3 are also present in the circulation following hepatocyte synthesis.

Whether synthesised locally or derived from the circulation, C3 is cleaved into C3b and C3a. The larger fragment C3b covalently binds to the target cell surface and is further degraded into C3dg and C3d fragments, which remain covalently anchored to the cell. Both C3 and C3b have a relatively short half-life in serum or on the membrane and C3b degrades within minutes on the plasma membrane of the affected cell. Thereafter C3d is relatively stable *in situ* and can be detected for several days^4^. In various organ models of IRI, these covalently bound products of C3 degradation, i.e. C3b and C3d, are associated with tissue injury^5–7^. Following the cleavage of C3 and subsequently C5 three types of complement activation products are formed: the tissue bound products C3b and C3d interact with specific complement receptors (CR1-CR4 and CRIg) on leukocytes to mediate immune adhesion; the soluble peptides fragments C3a and C5a, which engage with the respective complement receptors (C3aR and C5aR1) on infiltrating and parenchymal cells to stimulate cell activation; and membrane attack complex (C5b-9), which mediates pore formation and membrane injury. Therefore, C3d can be considered a ‘footprint’ formed early during complement activation and a potential marker of tissue injury in myocardial reperfusion damage.

Studies in other models of inflammatory and immune disease have shown close correlation between the accumulation of C3d, the duration of ischaemia and tissue infarction^5,6^. Moreover, there are numerous pre-clinical and clinical studies that demonstrated involvement of Complement and in particular C3d in myocardial reperfusion injury^8–11^. As a clinical assay, complement measurement in blood, urine and tissue biopsy samples is widely used as a biomarker for many clinical conditions^12^. However in some cases these measurements have provided limited whole organ information and have often yielded false positive or false negative results, possibly since systemic measurements do not adequately reflect complement activation localised to within an organ and due to biopsy sampling error^13^. Currently there are no validated techniques to non-invasively assess the extent and location of molecular markers of tissue injury within an affected organ soon after IRI. Most existing imaging techniques rely on functional or anatomical measurements such as organ perfusion or scar formation, respectively, and are often performed several days or even months following the acute injury, with little value in prognosis. Reperfusion of ischaemic tissues may not always rescue the jeopardized tissue, and, particularly after longer periods of ischaemia and after reperfusion therapy, can cause additional tissue damage^14,15^. The extent to which complement contributes to myocardial infarct size in man is not fully known, though preliminary porcine and human studies have suggested that inhibition of complement activation may indeed limit infarct size^16,17^. It has also been shown that myocardial reperfusion therapy in humans who suffer and subsequently die from AMI is associated with significantly more complement deposition, suggesting that activation of complement in the ischaemic human myocardium may be enhanced by reperfusion^18^.

Non-invasive imaging to delineate C3d in tissues could prove an asset to inflammatory pathway detection, monitoring and targeting specific intervention. We have recently developed a Single Photon Emission Computed Tomography (SPECT) imaging agent, namely ^99m^Technecium-labelled recombinant Complement Receptor 2 (^99m^Tc-rCR2). The endogenous human CR2 (CD21) is normally expressed an antigen presenting cells and B cells. It is formed of 16 short consensus repeats (SCRs) and specifically binds to the degraded components of activated, membrane bound C3 fragments, namely iC3b, C3dg and C3d, on the surface of targeted cells^19^. The first two N-terminal domains, SCR1 and SCR2, are required for binding to C3d^20,21^. The C3d-binding domain of SCR1/2 is a 15.5 kDa recombinant human fragment (rCR2) which we have expressed, refolded and purified from *E. coli*^19^. The rCR2 fragment was expressed with a fused C-terminal histidine tag, both to facilitate purification and to allow site specific radiolabelling with the technetium-99m synthon [^99m^Tc(CO)_3_(OH_2_)_3_]^+^ ^22^ to from ^99m^Tc-rCR2. The function of the recombinant human CR2 was confirmed by binding specifically to rat, mouse and human C3d^+^. Human rCR2 is cross reactive with mouse and rat C3^19^.

In a previous study, we used a heterotopic cardiac transplant model in mice to examine the ability of ^99m^Tc-rCR2 to localise to global areas of myocardial IRI induced by cold ischaemia prior to implant surgery. By SPECT imaging, we found evidence of specific uptake within the injured myocardium corresponding to areas of complement deposition^23^.

Here we studied the left anterior descending coronary artery ligation model of myocardial IRI, to investigate the potential utility of ^99m^Tc-rCR2 imaging in a restricted area of myocardial injury following a short period normothermic, clinically relevant, ischaemia and serve as a basis for future studies of the inflamed but viable area of myocardial damage after revascularisation therapy in the clinic.

## Results

### ^99m^Tc-rCR2 SPCET/CT imaging specifically detects C3d in hearts post IRI

**Figure 1:**
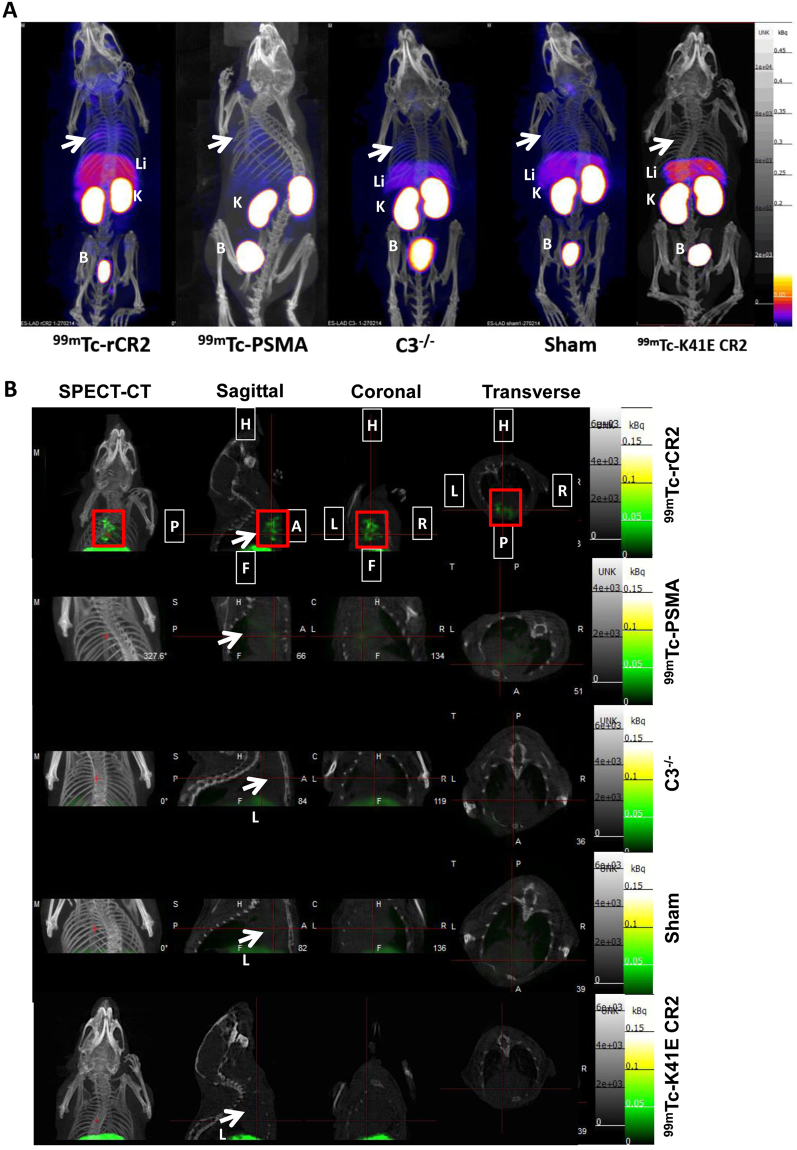
C3d is detected by ^99m^Tc-rCR2 imaging using NanoSPECT-CT. Following the IRI or sham procedures, mice received an intravenous injection of ^99m^Tc-rCR2, ^99m^Tc-PSMA or ^99m^Tc-K41E CR2. After 1hr, the mice underwent imaging for 45 min using NanoSPECT-CT. Signals were present in the liver [Li], Kidneys [K] and bladder [B]. The white arrow indicates the location of the heart. (**A**) Whole body SPECT-CT imaging of BL/6 or C3^−/−^ mice injected with ^99m^Tc-rCR2, ^99m^Tc-PSMA or ^99m^Tc-K41E CR2. (**B**) SPECT-CT, Sagittal, Coronal and Transverse images of the region of the infarcted hearts, H = Head, F = Feet, L = Left, R = Right, P = Posterior and A = Anterior. Focal uptake shown in red box.

**Figure 2:**
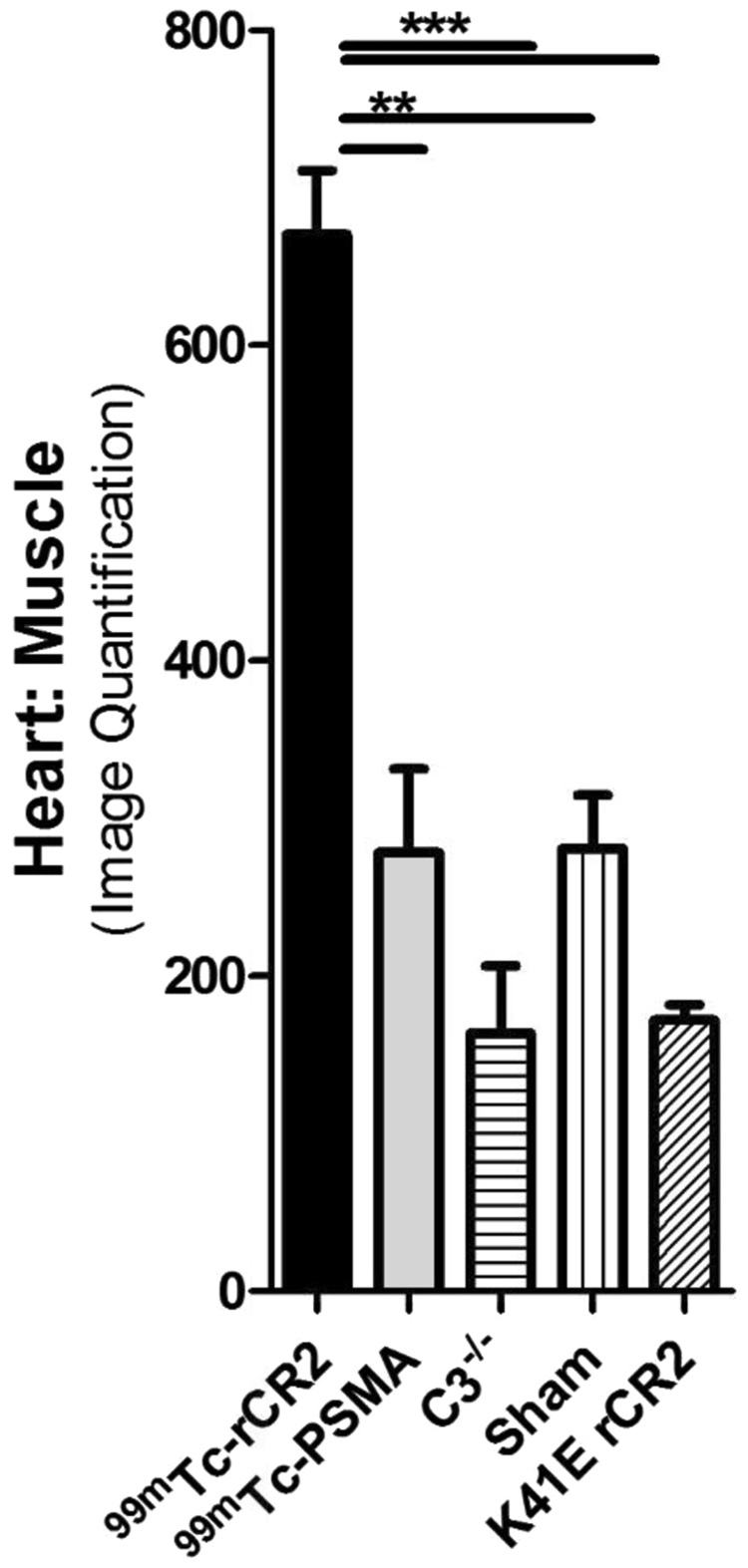
Biodistribution studies confirming presence of ^99m^Tc-rCR2 in the post-ischaemic hearts. Quantification of SPECT-CT images for levels of radioactivity in the heart post IRI induction is shown. The heart signals were corrected to skeletal muscle (thighs) signals. P of <0.01 and <0.0001 are labelled as ** and *** respectively.

To study the extent of complement activation in injured heart muscle, we used radiolabelled rCR2 to non-invasively image complement activation product after induction of IRI in a mouse model^24,25^. We used wild type (C3^+/+^) or C3 deficient (C3^−/−^) mice for the study. We induced IRI by the left anterior descending coronary artery ligation with a fixed ischaemic time (30 minutes), except for mice that underwent a sham procedure consisting of surgery and no coronary artery ligation. 24 hrs after surgery, the mice received either ^99m^Tc-rCR2 or control imaging ligand. The five initial groups of mice studied were: C3^+/+^ mice injected with ^99m^Tc-rCR2 (Group 1); C3^+/+^ mice injected with irrelevant protein ^99m^Tc-PSMA (Group 2); C3^−/−^ mice injected with ^99m^Tc-rCR2 (Group 3); sham treated C3^+/+^ mice injected with ^99m^Tc-rCR2 (Group 4); and C3^+/+^ mice treated with inactive rCR2 mutant ^99m^Tc-K41E CR2 (Group 5). Results of SPECT/CT imaging starting at 1 hour after injection of Tc-labelled tracer injection and continuing for 45 minutes are shown in Fig. 1A and B and Supplementary Fig. 2a and b. These show ^99m^Tc-rCR2 uptake in the myocardium (position indicated by white arrows) of Group 1 mice, but not in control studies in Groups 2–5 mice. Quantification of the SPECT/CT images showed significantly higher uptake in Group 1 (645.9 ± 46.0 ratio of heart to skeletal muscle) as compared to Group 2 (278.0 ± 53.2 ratio of heart to skeletal muscle, *p* = *0.0032*), Group 3 (163.5 ± 43.0 ratio of heart to skeletal muscle, *p* = *0.0008*), Group 4 (280.5 ± 34.1 ratio of heart to skeletal muscle, *p* = *0.0016*) or Group 5 (172.0 ± 9.8 ratio of heart to skeletal muscle, *p* = *0.0001*) (Fig. 2) (p = 0.0003).

All five groups demonstrated high uptake of the respective tracer in the kidney (Group 1, 37.6 ± 7.9; Group 2, 30.3 ± 7.5; Group 3, 113.1 ± 10.9 and Group 4, 19.7 ± 7.8 percent injected dose per gram per blood [%ID/g], Supplementary Fig. 3b) and bladder due to the non-specific retention and clearance of the radiolabelled protein (Fig. 1 and Supplementary Fig. 2). In addition, the groups that received ^99m^Tc-rCR2 also showed some uptake in the liver (Group 1, 6.1 ± 0.9; Group 3, 8.2 ± 0.6 and Group 4, 2.8 ± 0.9%ID/g per blood, Supplementary Fig. 3b).

To confirm that the enhanced uptake of tracer in the heart was specific for ^99m^Tc-rCR2 and dependent on the presence of C3d in ischaemic tissue, we killed the mice after imaging and removed the organs for *ex vivo* biodistribution analysis histology. We weighed and measured the radioactivity of the organs and expressed the biodistribution of tracer as the heart to blood ratio of the injected dose/gram of tissue (%ID/g) (Supplementary Fig. 3). Group 1 hearts demonstrated significantly higher uptake (1.17 ± 0.11) as compared to Group 2, ^99m^Tc-PSMA, (0.35 ± 0.03), Group 3, C3^−/−^ (0.43 ± 0.05), or Group 4, Sham operated hearts (0.41 ± 0.05) (*p* < *0.0001*). Both *ex vivo* biodistribution and non-invasive image quantification confirmed the data obtained by non-invasive SPECT-CT image analysis of the hearts (Fig. 1 and Supplementary Fig. 2).

### Immunohistological confirmation of C3d expression in infarcted hearts

**Figure 3:**
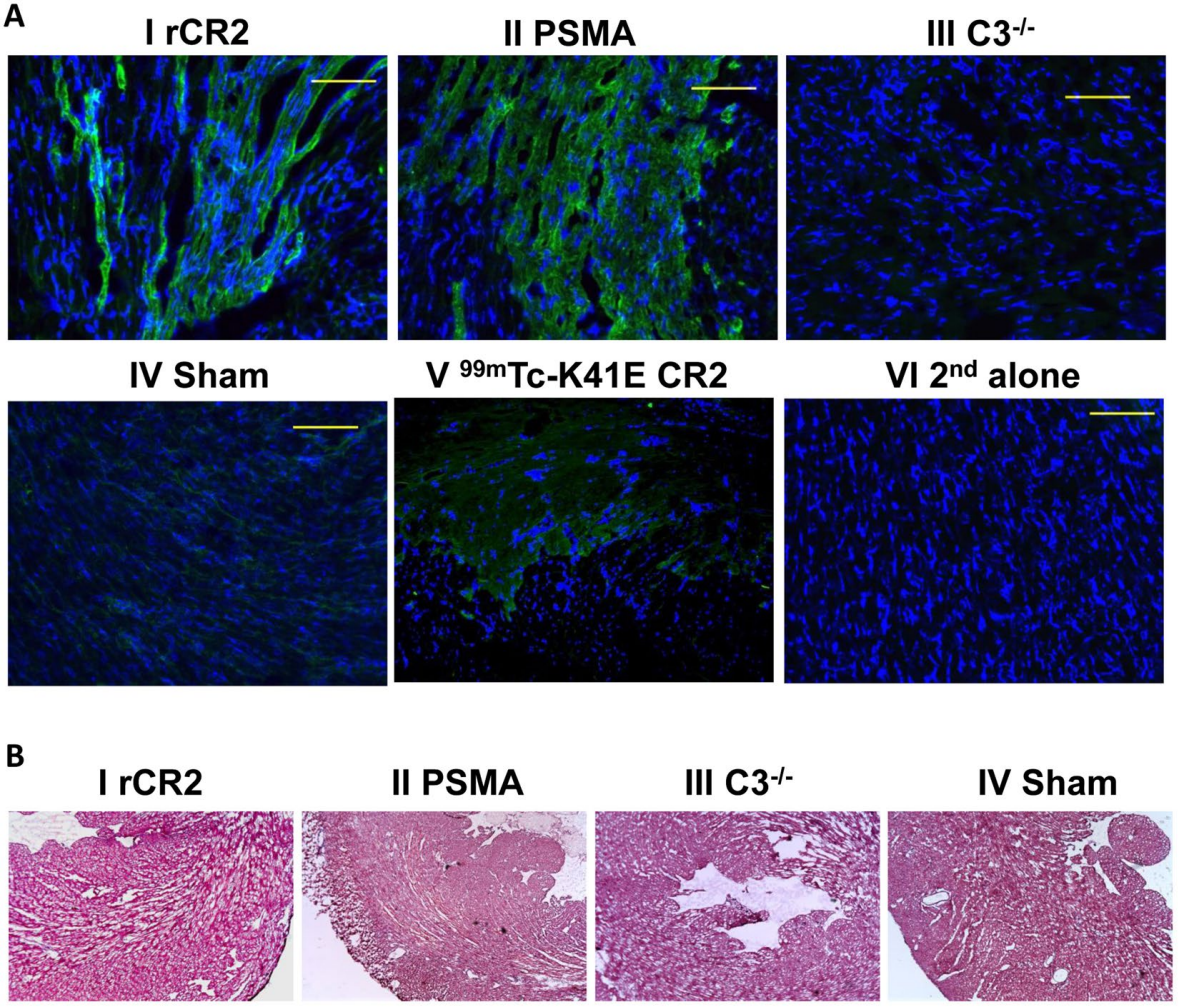
Histological studies demonstrating C3d expression in post-ischaemic heart. (**A**) After the imaging studies, hearts were removed, sectioned and stained with anti-C3d (green) and DAPI (blue). Representative sections shown are from mid region of the heart of BL/6 mice injected with ^99m^Tc-rCR2 (I) or ^99m^Tc-PSMA (II), and of C3^−/−^ mice injected with ^99m^Tc-rCR2 (III), or from sham operated BL/6 mice injected with ^99m^Tc-rCR2 (IV) or BL/6 mice injected with ^99m^Tc-K41E CR2 (V). Control BL/6 post-ischaemic tissue stained with secondary antibody alone is shown (VI). Magnification is ×40. (**B**) H & E staining of mid heart sections of the mice injected with ^99m^Tc-rCR2 (I), ^99m^Tc-PSMA (II), C3^−/−^ mice injected with ^99m^Tc-rCR2 (III) and sham operated mice injected with ^99m^Tc-rCR2 (IV).

We investigated the expression C3d in cardiac tissue to determine if the uptake of ^99m^Tc-rCR2 in SPTECT/CT imaging and *ex vivo* biodistribution studies (Fig. 1 and Supplementary Figs 2 and 3) corresponded to the immunohistological detection of C3d with anti-C3d antibody (Fig. 3A). The expression of C3d was evident in the hearts of Group 1 (^99m^Tc-rCR2), Group 2 (^99m^Tc-PSMA) and Group 5 (^99m^Tc-K41E CR2), but not of Group 3 (C3^−/−^) or Group 4 (sham). Hence there was good correspondence between the image obtained with radiolabelled CR2 and histological detection of C3d in the heart, but no inappropriate detection in images where C3d was absent from tissues (Groups 3 and 4) or where irrelevant imaging agent was used despite the presence of C3d (Groups 2 and 5) No significant morphological differences between the groups were observed in heart sections stained with H & E (Fig. 3B).

### Treatment with complement regulator Crry-Ig reduces the cardiac imaging signal with ^99m^Tc-rCR2 SPECT/CT

**Figure 4:**
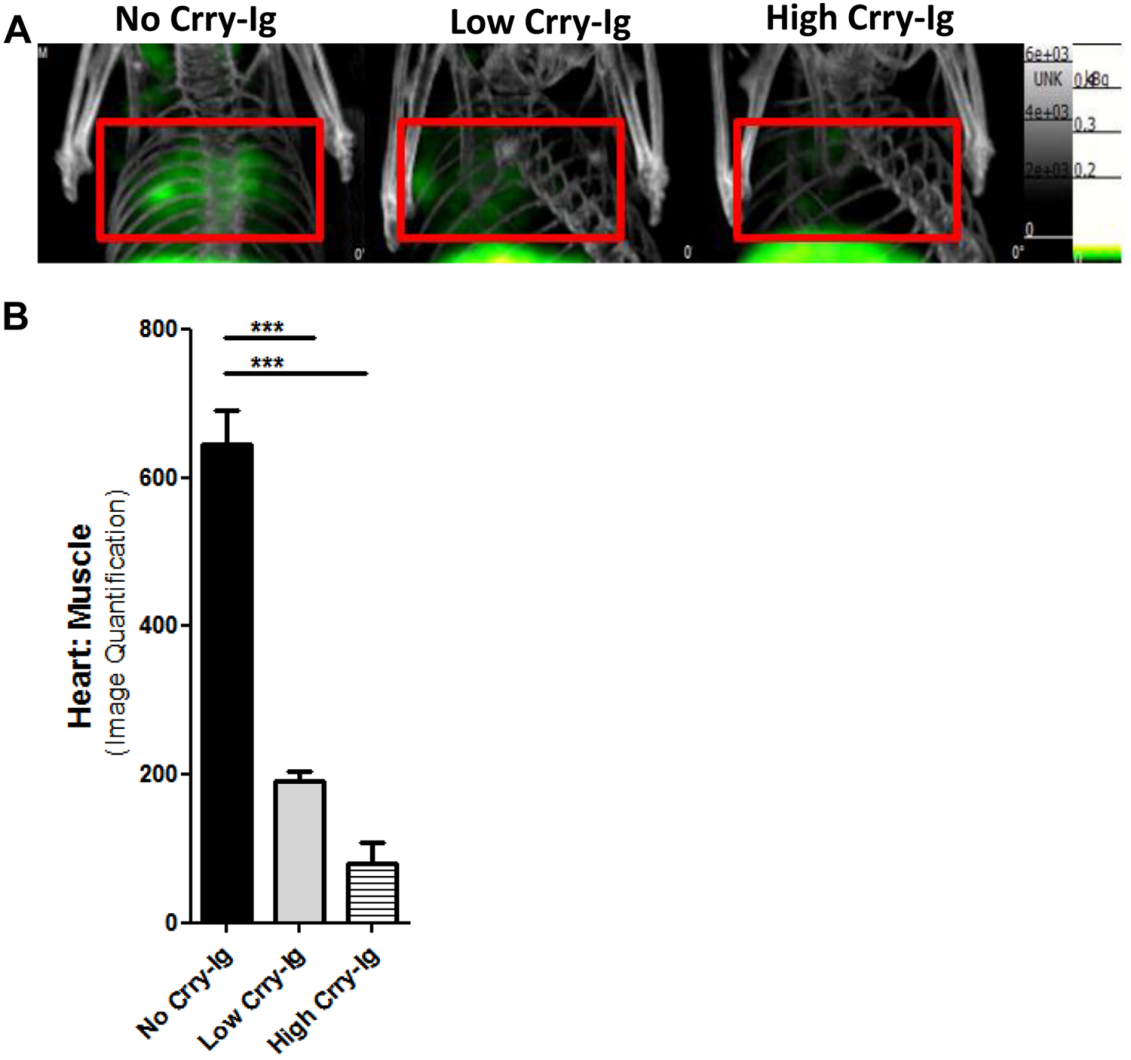
^99m^Tc-rCR2 SPCET/CT imaging detects different levels of activated myocardial complement (C3d) *in vivo*. Mice underwent induction of IRI in the presence of presence of complement inhibitor Crry-Ig, administered intravenously in low (0.75 mg) or high (1.0 mg) doses prior to IRI induction. (**A**) Representative images after administration of ^99m^Tc-rCR2, indicating the C3d marker is reduced in strength according to dose of Crry-Ig. (**B**) Imaged-based quantification of the heart signal is shown.

Previous studies have found therapeutic inhibition of C3 cleavage to protect the heart against induction of IRI. To determine whether imaging with ^99m^Tc-rCR2 SPECT/CT can detect changes in complement deposition due to therapeutic intervention we used the CR1-related gene/protein y-Ig (Crry-Ig)^26^. Crry-Ig is known to inhibit the e classical and alternative pathway C3 convertases and thus prevent the formation of C3b and thus inhibit C3d generation. We injected C3^+/+^ mice i.v. with either 0.75 mg (Group 6) or 2 mg (Group 7) doses of Crry-Ig^26^ prior to the induction of IRI. Following the induction of IRI, the two treatment groups were then imaged with ^99m^Tc-rCR2 and the results compared to the untreated group of mice (Group 1) of the earlier study. As shown in Fig. 4A, the heart signals observed in both low and high dose treatment groups (Groups 6 and 7) were lower than in the untreated group (Group 1). Image-based quantification of the heart signal demonstrated a significant difference between the signal in Group 1 (645.9 ± 46.02) compared to Group 6 (191.7 ± 13.28) and Group 7 (80.60 ± 27.39) treatment groups (*p* = *0.0002*) (Fig. 4B). *Ex vivo* organ biodistribution studies also confirmed significant difference between no treatment Group 1 (1.17 ± 0.11) and low (0.48 ± 0.13) and high (0.34 ± 0.03) dose treatment groups 6 and 7 respectively (*p* = *0.0082*) (Supplementary Fig. 4).

## Discussion

Our results demonstrate the capability of SPECT imaging combined with radiolabelled CR2 to detect *in vivo* complement C3 deposited the myocardium after the induction of cardiac IRI. Control imaging studies showed that the uptake of radiolabelled tracer in ischaemic myocardium was specific for the imaging ligand ^99m^Tc-rCR2 and found no major uptake using a mutated rCR2 protein or an irrelevant protein (PSMA antibody), or when the specific tracer was applied to coronary artery ligation studies in C3^−/−^ mice or to mice that underwent a sham procedure. The capacity of SPECT imaging combined with ^99m^Tc-rCR2 to detect differences in tissue levels of C3d following therapeutic complement inhibition was also apparent in our results, indicating the sensitivity of the imaging method to quantify dose-dependent changes in C3d generation in the injured myocardium. In addition, through *ex vivo* biodistribution and histological studies, we validated our findings with *in vivo* imaging. These data suggest that the imaging method using ^99m^Tc-rCR2 can provide specific and sensitive information regarding the deposition of complement in post-ischaemic myocardium. Furthermore, ^99m^Tc-rCR2 coupled with SPECT imaging could be a promising method to monitor the inflammatory status of myocardial tissue damaged by IRI. This is of particular interest regarding the area of inflamed tissue outside the infarct that is potentially viable.

Mechanism-based approaches that have entered clinical practice to reduce damage progression after AMI are few. IRI involves direct cardiomyocyte death and myocardial stunning, arrhythmias and the “no reflow” phenomenon^27^. Several approaches including pre-, post-, and remote-ischemic conditioning and several pharmacological interventions including immune modulation have shown promise in both pre-clinical and clinical models^28^. However, although the treatment of acute coronary heart disease has been sustainably improved with the introduction of percutaneous coronary intervention, there is currently no widely accepted or specific therapy that addresses this important clinical problem^27^. Experimental and clinical studies strongly implicate the complement system as pivotal in IRI^8,10,11,29^ and therapeutic depletion or inhibition of complement at the level of C3 or C5 has been found to reduce myocardial infarct size in different animal models^30,31^. Nonetheless, the results from large trials using an anti-C5 antibody have thus far been disappointing^27^. While there was significant reduction in mortality using complement C5 inhibition in the setting of CABG the results of anti-C5 therapy in patients with AMI undergoing percutaneous coronary intervention showed no significant benefit^32^. A non-invasive biomarker to directly quantify the concentration of activated complement in injured myocardium could be a valuable tool in defining which patients are likely to benefit from complement-directed therapy. Such a tool would also help to monitor the response to anti-complement treatment and provide a marker of effectiveness with different therapeutic agents.

In this study, we used a short ischaemic time in order to minimise the degree of cardiac necrosis but allow the generation of complement activation product in viable tissue. This is in contrast with some other studies that have used longer ischaemia times (e.g. 24 hours) to study myocardial infarction^33^. As a result, we found that the imaging signal was clearly sensitive to distinguish ischaemic from non-ischaemic tissues and discriminate between different levels of tissue complement product (C3d) associated with therapeutic intervention. In addition, the short ischaemic time employed in this study allowed us to show that C3d detection by this method can be independent of myocardial necrosis. In this respect, ^99m^Tc-rCR2 should be useful for future studies aiming to delineate inflamed but viable tissue outside the region of the infarct.

In conclusion, the results presented here support the development of labelled CR2 as an imaging ligand of choice, since it gives highly specific detection of C3d corresponding to the area of myocardial injury and is sufficiently sensitive to detect different degrees of C3d present by external body imaging. This method for non-invasive assessment of C3d in injured myocardial tissue has potential use for quantifying the extent of complement mediated damage, in stratifying patient groups for possible treatments with complement inhibitors and in assessing the response to such therapies. This may be particularly important since some therapeutic approaches have failed to deliver on earlier promise in large scale clinical studies; and it is possible that patient selection according to demonstrable complement involvement could lead to better recognition of homogenous patient groups for inclusion in clinical trials and for early detection of treatment efficacy in relation to long term clinical outcome. This study therefore opens the door for translational studies in man evaluating the utility of complement imaging techniques in future biomarker/biotherapy studies. And, to facilitate such studies, although the present Tc-labelled tracer is a start, a PET ligand (e.g. Ga-68 or F-18) may be superior in resolving localised regions in the myocardium and also quantifying them, compared to SPECT.

## Materials and Methods

### Mice and induction of ischaemia-reperfusion injury

All studies used age (6–8 weeks old) and sex (female) matched C57BL/6 (referred as BL/6, purchased from Harlan) or C57BL/6.C3^−/−^ mice (derived by homologous recombination in embryonic stem cells and backcrossed on to BL/6 parental strain for at least 10 generations^34,35^). Housing conciliations were specific pathogen free and all experiments proceeded in accordance with national guidelines for animal care. Killing the mice at the end of the experiments involved dislocation of the neck. Animal studies were carried out in accordance with UK Research Councils’ and Medical Research Charities’ guidelines on Responsibility in the Use of Animals in Bioscience Research, under a UK Home Office license.

The induction of myocardial IRI used temporary occlusion (30 min) and reperfusion of the left anterior descending coronary artery, and sham procedures consisted of surgery without coronary artery occlusion (Supplementary Fig. 1) as described previously^24,25^.

Imaging studies used the NanoSPECT/CT on the day after the IRI induction procedure, following an i.v. injection of approximately 60 MBq of radiotracer (see below). There were five groups of mice in the initial IRI study: C3^+/+^ mice injected with ^99m^Tc-rCR2 (Group 1, n = 8); C3^+/+^ mice injected with irrelevant protein ^99m^Tc-PSMA (Group 2, n = 3); C3^−/−^ mice injected with ^99m^Tc-rCR2 (Group 3, n = 3); sham processed C3^+/+^ mice injected with ^99m^Tc-rCR2 (Group 4, n = 6) and C3^+/+^ mice treated with inactive rCR2 mutant ^99m^Tc-K41E CR2 (Group 5, n = 3).

### Peptide radiolabelling with ^99m^Tc

We previously reported developing a 15.5 kDa peptide which consisted of Short Consensus Repeats 1 and 2 of Complement Receptor 2 (CR2), the binding domain for C3d^36^. The peptide was engineered to include the C-terminal sequence VFPLECHHHHHH, a hexahistidine tag for site-specific radiolabelling with [^99m^Tc(CO)_3_(OH_2_)_3_]^+^ (^99m^Tc-Tricarbonal)^19^. Briefly, ^99m^Tc pertechnetate eluted with saline from a Drytec generator (GE Healthcare, Amersham, UK) was converted to [^99m^Tc(CO)_3_(OH_2_)_3_]^+^ using a kit from Isolink kit (Covidien, Petten, The Netherlands). Proteins were labelled with ^99m^Tc by incubating 100 µg of rCR2 or PSMA in 100 µL of PBS pH 7.4 with an additional 350 mM NaCl and up to 600 MBq of [^99m^Tc(CO)_3_(OH_2_)_3_]^+^ in 100 µL at 37 °C. Then 20 µg of radiolabelled peptides where intravenously injected via tail vein.

### NanoSPECT/CT *in vivo* Imaging

Mice received an injection of 100 µL ^99m^Tc-rCR2 (20 µg of rCR2) or 100 µL ^99m^Tc-PSMA (20 µg of PSMA) into via the tail vein, followed after 1 hour by single photon emission tomography imaging. This used a small-animal NanoSPECT/CT with silver upgrade (Mediso, Budapest, Hungary). The mice were under isofluorane anaesthesia and respiration monitoring, and a heating pad was in use to ensure constant body temperature of 37 °C during imaging. Image analysis employed VivoQuant software (inviCRO, Boston, USA).

### *Ex vivo* organ biodistribution and histological studies

Following imaging, mice were killed and organs removed and weighed. To determine the presence of ^99m^Tc, the removed organs underwent gamma counting using a multi well automated Wallac 1282 Compugamma Universal Gamma Counter (LKB Wallac, PerkinElmer, Cambridge, UK). Biodistribution data were expressed as the ratio of heart to blood injected dose/gram (ID/g).

For histological studies, we used heart samples covered with optimal cutting temperature bedding compound (VWR Chemicals, Leuven, Belgium) and snap-frozen in liquid nitrogen before storing at −80 °C. We prepared sections of the mid heart region (transverse axis) at 5 µm thicknesses using 5030 cryostat (Bright, Cambridgeshire, UK) and transferred them to polylysene coated glass slides. We then fixed the specimens by incubation with acetone at 4 °C for 5 minutes, followed by washing the slides were with PBS and blocking with 20% foetal bovine serum (FBS, Life technologies) in PBS for 30 minutes at room temperature. We then incubated the slides with rabbit anti-C3d (ab15981, Abcam, UK) at 1/200 dilution for 1 hr at room temperature, and for a further 30 mins at room temperature with secondary antibody goat anti-Rabbit Dylight 488 (ab96895, Abcam, UK) at 1/1000 dilution. We then washed the slides and added DAPI mounting serum (ProLong Gold, Invitrogen) to co-stain for nuclei. We examined an prepared images of the slides using DM6000B fluorescent microscope (Leica, Wetzlar, Germany).

For Hematoxylin and Eosin staining, we fixed frozen coronal mid heart sections (10 μm) in 4% paraformaldehyde for 45 min at room temperature followed by washing in distilled water. We then incubated the tissue sections with Harris-modified haematoxylin solution (Sigma Aldrich) for 2 min and rinsed with water. We subsequently differentiated the tissue sections in 1% acid alcohol solution (70% ethanol, concentrated HCl) and washed with water. Counterstaining was with eosin Y aqueous solution (Sigma Aldrich) for 30 seconds followed by rinsing with water. Incubation of the sections in 70% ethanol (1 min), 95% ethanol (1 min) and Histo-Clear II solution (National Diagnostics, 1 min, 2 changes) then followed. Finally, we mounted the tissue sections using DPX new mounting medium (Merck Millipore) under cover slips. We evaluated the H&E stained sections under bright-field microscopy using a light microscope (DM6000 B; Leica Microsystems Ltd., Bucks, UK).

### Crry-Ig Treatment

To inhibit complement activation and thus reduce C3d formation, we used the C3 convertase inhibitor CR1-related gene/protein y-Ig (Crry-Ig), which inhibits both the classical and alternative pathways of complement activation^26^. C3^+/+^ mice received intravenous injection of Crry-Ig in two groups, low and high dose. Low dose consisted of a single 0.75 mg immediately before induction of IRI (Group 6, n = 3). High dose consisted of a 1 mg injection given one day before and immediately before IRI induction (Group 7, n = 3). The chosen doses were based on previous study^26^.

### Statistics

All analyses are reported as mean ± standard error of the mean (SEM). Data were analysed using GraphPad Prism Version 5 and statistical analysis were performed using one-way ANOVA and statistical significance assigned for p values < 0.05. If the one-way ANOVA was significant, then a post hoc analysis was performed with Dunnet’s test; p values of <0.05 were considered significant. Significant differences of <0.05 was labelled *, <0.01 was labelled ** and <0.001 was labelled ***.

## Electronic supplementary material

Supplementary Information

Supplementary Figure 2 video
