# Author Correction: Non-Invasive whole-body detection of complement activation using radionuclide imaging in a mouse model of myocardial ischaemia-reperfusion injury

**DOI:** 10.1038/s41598-018-22722-x

**Published:** 2018-03-13

**Authors:** Ehsan Sharif-Paghaleh, May Lin Yap, Sarah-Lena Puhl, Adam Badar, Julia Baguña Torres, Krisanat Chuamsaamarkkee, Florian Kampmeier, Richard A. Smith, James Clark, Philip J. Blower, Steven Sacks, Gregory E. Mullen

**Affiliations:** 1 Division of Imaging Sciences and Biomedical Engineering, St Thomas’ Hospital, King’s College London, London, UK; 2 MRC Centre for Transplantation, King’s College London, London, UK; 3 Department of Immunology, School of Medicine, Tehran University of Medical Sciences, Tehran, Iran; 4 Cardiovascular Division, Faculty of Life Sciences and Medicine, King’s College London, London, UK

Correction to: *Scientific Reports* 10.1038/s41598-017-16387-1, published online 23 November 2017

The Acknowledgements section in this Article is incomplete.

“The authors acknowledge the financial support from King’s College London, Medical Research Council Centre of Transplantation, the British Heart Foundation, the Centre of Excellence in Medical Engineering funded by the Wellcome Trust and the EPSRC under grant number WT088641/Z/09/Z, the King’s College London and University College London Comprehensive Cancer Imaging Centre funded by CRUK and EPSRC in association with the MRC and DoH (England), and the National Institute for Health Research (NIHR) Biomedical Research Centre based at Guy’s and St Thomas’ NHS Foundation Trust and King’s College London. PET/CT scanning equipment was funded by the Wellcome Trust. JBT was supported by the Alzheimer’s Society We also thank Dr Kavitha Sunassee and Mr Stephen Clark for expert assistance with preclinical imaging. The views expressed are those of the authors and not necessarily those of the NHS, the NIHR or the Department of Health.”

should read:

“The authors acknowledge the financial support from King’s College London, Medical Research Council Centre of Transplantation, the British Heart Foundation [PG/11/111/29270], the Centre of Excellence in Medical Engineering funded by the Wellcome Trust and the EPSRC under grant number WT088641/Z/09/Z, the King’s College London and University College London Comprehensive Cancer Imaging Centre funded by CRUK and EPSRC in association with the MRC and DoH (England), and the National Institute for Health Research (NIHR) Biomedical Research Centre based at Guy’s and St Thomas’ NHS Foundation Trust and King’s College London. PET/CT scanning equipment was funded by the Wellcome Trust. JBT was supported by the Alzheimer’s Society We also thank Dr Kavitha Sunassee and Mr Stephen Clark for expert assistance with preclinical imaging. The views expressed are those of the authors and not necessarily those of the NHS, the NIHR or the Department of Health.”

