# Supplementary material for: Non-Invasive whole-body detection of complement activation using radionuclide imaging in a mouse model of myocardial ischaemia-reperfusion injury
Source: Sci Rep. 2017 Nov 23;7:16090. doi: 10.1038/s41598-017-16387-1 (PMC5700950; doi:10.1038/s41598-017-16387-1)

Supplementary Information

Non-Invasive whole-body detection of complement activation using radionuclide imaging in a mouse model of myocardial ischaemia-reperfusion injury

Authors:

Ehsan Sharif-Paghaleh[1,2,3], May Lin Yap[1¬], Sarah-Lena Puhl[3¬], Adam Badar[1], Julia Baguña Torres[1], Krisanat Chuamsaamarkkee[1], Florian Kampmeier[1], Richard A.Smith[2], James Clark[4], Philip J. Blower[1], Steven Sacks[2*] and Gregory E. Mullen[1&2*]

¬: equal second authorship

*: equal last authorship

Author Affiliation:

[1] Division of Imaging Sciences and Biomedical Engineering, St Thomas' Hospital, King's College London, UK

[2] MRC Centre for Transplantation, King's College London, UK

[3] Department of Immunology, School of Medicine, Tehran University of Medical Sciences, Tehran, Iran

[4] Cardiovascular Division, Faculty of Life Sciences and Medicine, King's College London, UK

Contact Information*:

Prof Steven Sacks (steven.sacks@kcl.ac.uk)

MRC Centre for Transplantation, Guy's Hospital, Faculty of Life Sciences and Medicine, King's College London, England

Dr Greg Mullen (greg.mullen@kcl.ac.uk)

Division of Imaging and Biomedical Engineering, St Thomas' Hospital, Faculty of Life Sciences and Medicine, King's College London, England

Figure S1: Myocardial IRI model setup.

Figure S2: SPECT-CT movie of imaging complement protein by ^{99m}Tc-rCR2. Images are shown for mice that underwent induction of IRI followed by injection of ^{99m}Tc-rCR2 or the control peptides ^{99m}Tc-PSMA or ^{99m}Tc-K41E CR2, 1 hour prior to imaging. Images acquired using using NanoSPECT-CT. (A) Whole body SPECT-CT imaging of BL/6 or C3$^{-/-}$ mice injected with ^{99m}Tc-rCR2 or ^{99m}Tc-PSMA or ^{99m}Tc-K41E CR2. (B) SPECT-CT movies of the chest region of mice.

Figure S3: *Ex vivo* biodistribution studies validating the presence of ^{99m}Tc-rCR2 in the post-ischaemic hearts. After imaging studies, mice were culled and the hearts (**a**) and the rest of organs (**b**) were removed, weighed and subjected to *ex vivo* biodistribution analysis.

Figure S4: *Ex vivo* organ biodistribution study following inhibition of complement activation and consequent C3d formation by Crry-Ig. After imaging studies, mice injected with Crry-Ig to inhibit complement activation and hence reduce C3d formation were culled and hearts were removed and weighed and gamma-counted. Biodistribution data were expressed as the heart to blood ratio.

Supplementary Information

Fig 1

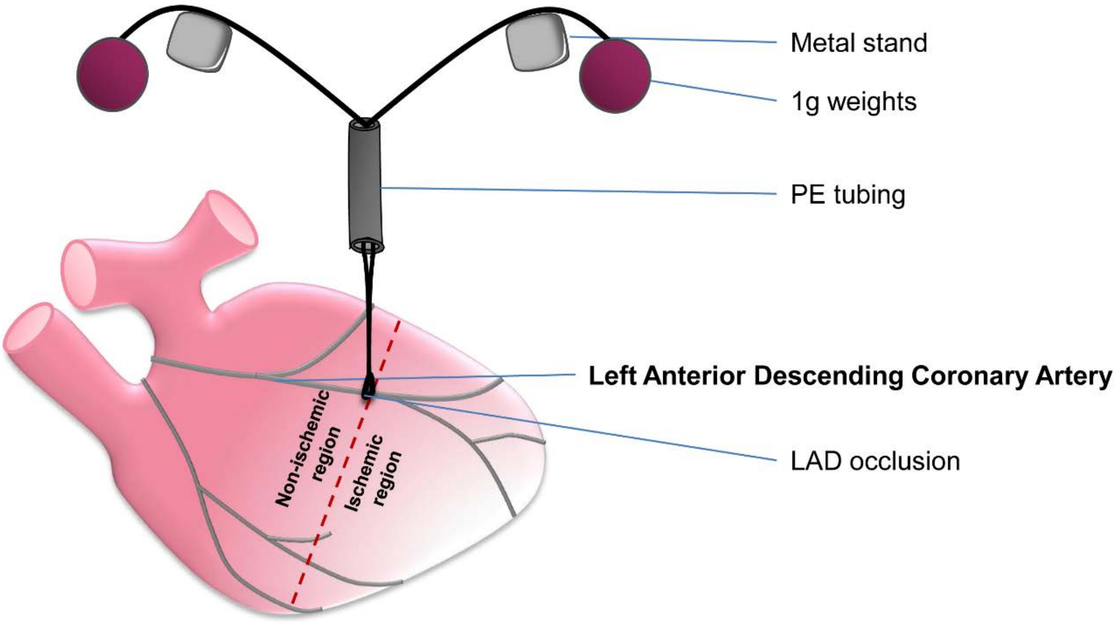

Video:

Fig 2

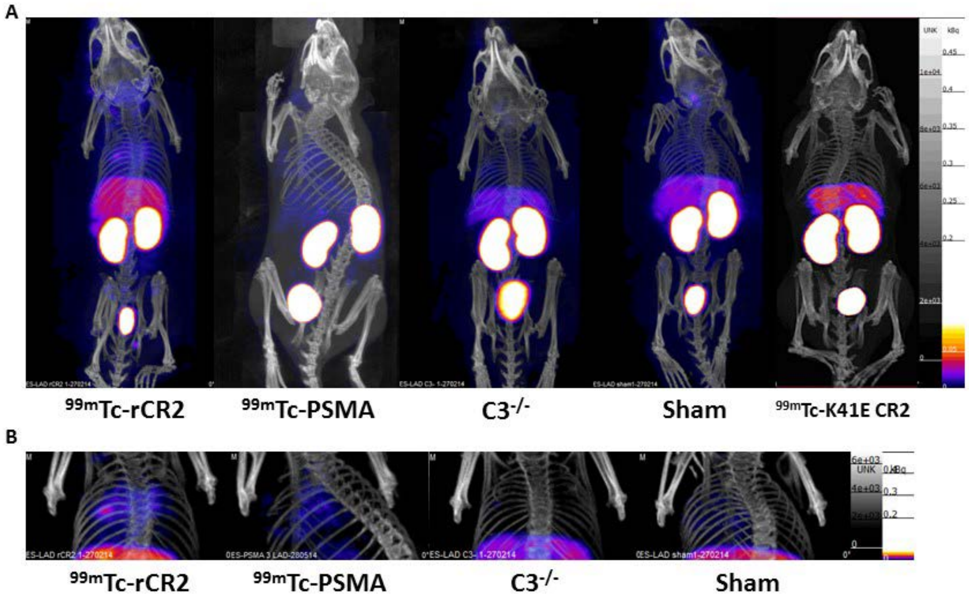
A
99mTc-rCR2
99mTc-PSMA
C3-/-
Sham
99mTc-K41E CR2
B
99mTc-rCR2
99mTc-PSMA
C3-/-
Sham

Fig 3

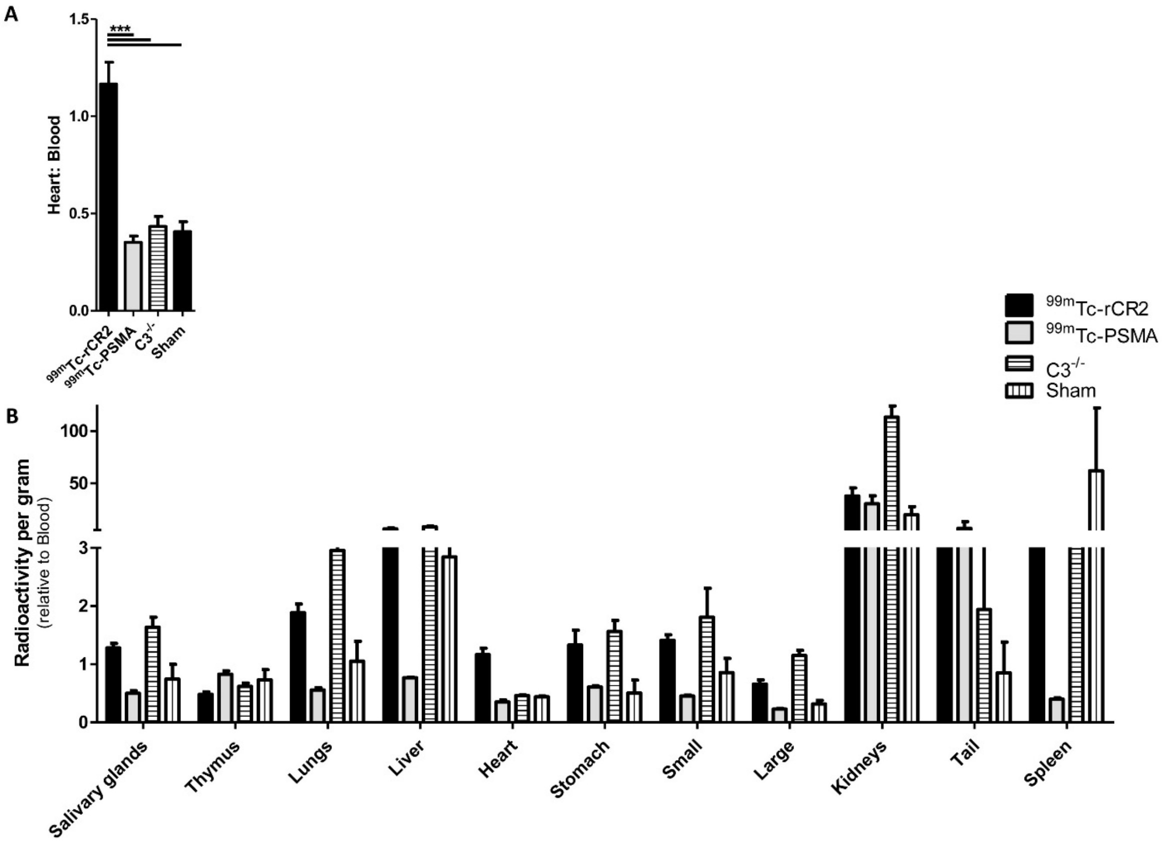
A
1.5
1.0
0.5
0.0
Heart: Blood
***
99mTc-rCR2
99mTc-PSMA
C3-/-
Sham
99mTc-rCR2
99mTc-PSMA
C3-/-
Sham
B
100
50
3
2
1
0
Radioactivity per gram
(relative to Blood)
Salivary glands
Thymus
Lungs
Liver
Heart
Stomach
Small
Large
Kidneys
Tail
Spleen

Fig 4

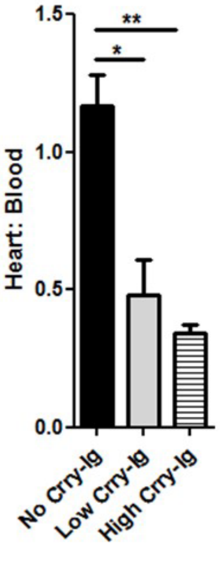

Supplement: Supplementary file 1 — Supplementary Information [file 41598_2017_16387_MOESM1_ESM.pdf]
